# An interdomain hydrogen bond in the Rag GTPases maintains stable mTORC1 signaling in sensing amino acids

**DOI:** 10.1016/j.jbc.2021.100861

**Published:** 2021-06-09

**Authors:** Shawn B. Egri, Kuang Shen

**Affiliations:** Program in Molecular Medicine, University of Massachusetts Medical School, Worcester, Massachusetts, USA

**Keywords:** mTOR complex 1 (mTORC1), Rag GTPase, enzyme mechanism, amino acid, hydrogen bond, nutrient sensing, co-IP, coimmunoprecipitation, CRD, C-terminal roadblock domain, mTORC1, mechanistic target of rapamycin complex 1, NBD, nucleotide-binding domain

## Abstract

Cellular growth and proliferation are primarily dictated by the mechanistic target of rapamycin complex 1 (mTORC1), which balances nutrient availability against the cell’s anabolic needs. Central to the activity of mTORC1 is the RagA–RagC GTPase heterodimer, which under favorable conditions recruits the complex to the lysosomal surface to promote its activity. The RagA–RagC heterodimer has a unique architecture in that both subunits are active GTPases. To promote mTORC1 activity, the RagA subunit is loaded with GTP and the RagC subunit is loaded with GDP, while the opposite nucleotide-loading configuration inhibits this signaling pathway. Despite its unique molecular architecture, how the Rag GTPase heterodimer maintains the oppositely loaded nucleotide state remains elusive. Here, we applied structure–function analysis approach to the crystal structures of the Rag GTPase heterodimer and identified a key hydrogen bond that stabilizes the GDP-loaded state of the Rag GTPases. This hydrogen bond is mediated by the backbone carbonyl of Asn30 in the nucleotide-binding domain of RagA or Lys84 of RagC and the hydroxyl group on the side chain of Thr210 in the C-terminal roadblock domain of RagA or Ser266 of RagC, respectively. Eliminating this interdomain hydrogen bond abolishes the ability of the Rag GTPase to maintain its functional state, resulting in a distorted response to amino acid signals. Our results reveal that this long-distance interdomain interaction within the Rag GTPase is required for the maintenance and regulation of the mTORC1 nutrient-sensing pathway.

The mechanistic target of rapamycin complex 1 (mTORC1) pathway is responsible for monitoring the availability of nutrients (amino acids and glucose) and growth factors and determining if sufficient materials are available to proceed with cellular growth (1, 2). A prerequisite step in the activation of mTORC1 is facilitated by the RagA–RagC GTPase heterodimer (3, 4). When cellular amino acid concentrations are high, the RagA subunit is loaded with GTP and the RagC subunit is loaded with GDP, which recruits mTORC1 to the lysosomal surface (3), where its kinase activity is stimulated by another small GTPase, Rheb, which is only active when growth factors are abundant (5, 6). In contrast, the reversed nucleotide-loading state of RagA–RagC inhibits the localization of mTORC1 to the lysosome (3). Coactivation of mTORC1 by RagA–RagC and Rheb ensures that both nutrients and growth factors are present before proceeding with downstream anabolic processes.

The architecture of the Rag GTPases is unique compared with canonical signaling GTPases such as Ras, as each functional unit of the Rag GTPases always consists of two subunits, RagA or RagB, bound to RagC or RagD. An individual Rag GTPase consists of a nucleotide-binding domain (NBD) that belongs to the Arf family GTPases and a C-terminal roadblock domain (CRD) that mediates heterodimerization (7, 8, 9, 10). The dimerized architecture establishes complicated conformational space for the Rag GTPase heterodimer. Upon GTP binding to a single subunit, the NBD of the Rag GTPase undergoes dramatic local conformational changes, as the Switch I motif swings to the top of the nucleotide-binding pocket and forms a lid (9, 10). In contrast, when the Rag GTPase binds to GDP, Switch I adopts an alpha-helical conformation where it extends away from the nucleotide-binding pocket and points toward the CRD (9, 10). Besides the local conformational changes, global conformational changes are coupled subsequently. As the NBD of the Rag GTPase is connected to the CRD through a flexible hinge, the relative position of the NBDs on the different subunits depends on the nucleotide-loading states, mutations, and interaction with binding partners. This global conformation change is critical to ensure precise recruitment or release of mTORC1.

Considering the complicated conformational space, recent studies have shown that intersubunit crosstalk maintains the nucleotide-loading state of the Rag GTPase heterodimer (11). Upon the binding of a GTP molecule to one subunit, it prevents GTP binding to the second subunit. In addition, the rate of GTP hydrolysis on the second subunit is increased significantly in the case that GTP binding were to occur. These mechanisms prevent the heterodimer from becoming dually loaded with GTP. Although the RagA–RagC heterodimer can theoretically occupy four varying nucleotide-loading states, only two of these states are functional, with each subunit only ever being loaded with a nucleotide opposite to that of the second subunit. This observation is also consistent with the functional output as the Rag GTPase heterodimer exerts a strongest phenotype in conducting amino acid signals when the two subunits are loaded with opposite nucleotides.

Despite the advances in understanding the molecular mechanism through which the Rag GTPases function, it is unclear how the Rag GTPases maintain their oppositely nucleotide-loaded state at the molecular level. In this study, we tackled this question by investigating a hydrogen bond formed between the NBD and CRD only in the GDP-loaded state. This interdomain interaction is evolutionarily conserved and impairing it causes major changes in the behavior of the Rag GTPase heterodimer, in which the GDP-loaded state is destabilized and the Rag GTPases fail to secure the oppositely nucleotide-loaded states. Furthermore, we show that when disrupted *in vivo*, cells lose the ability to respond effectively to changes in amino acid availability. Our results reveal a critical interdomain interaction that is essential in the process of amino acid signal transduction.

## Results and discussion

### Identification of an interdomain hydrogen bond in the Rag GTPases

**Figure 1:**
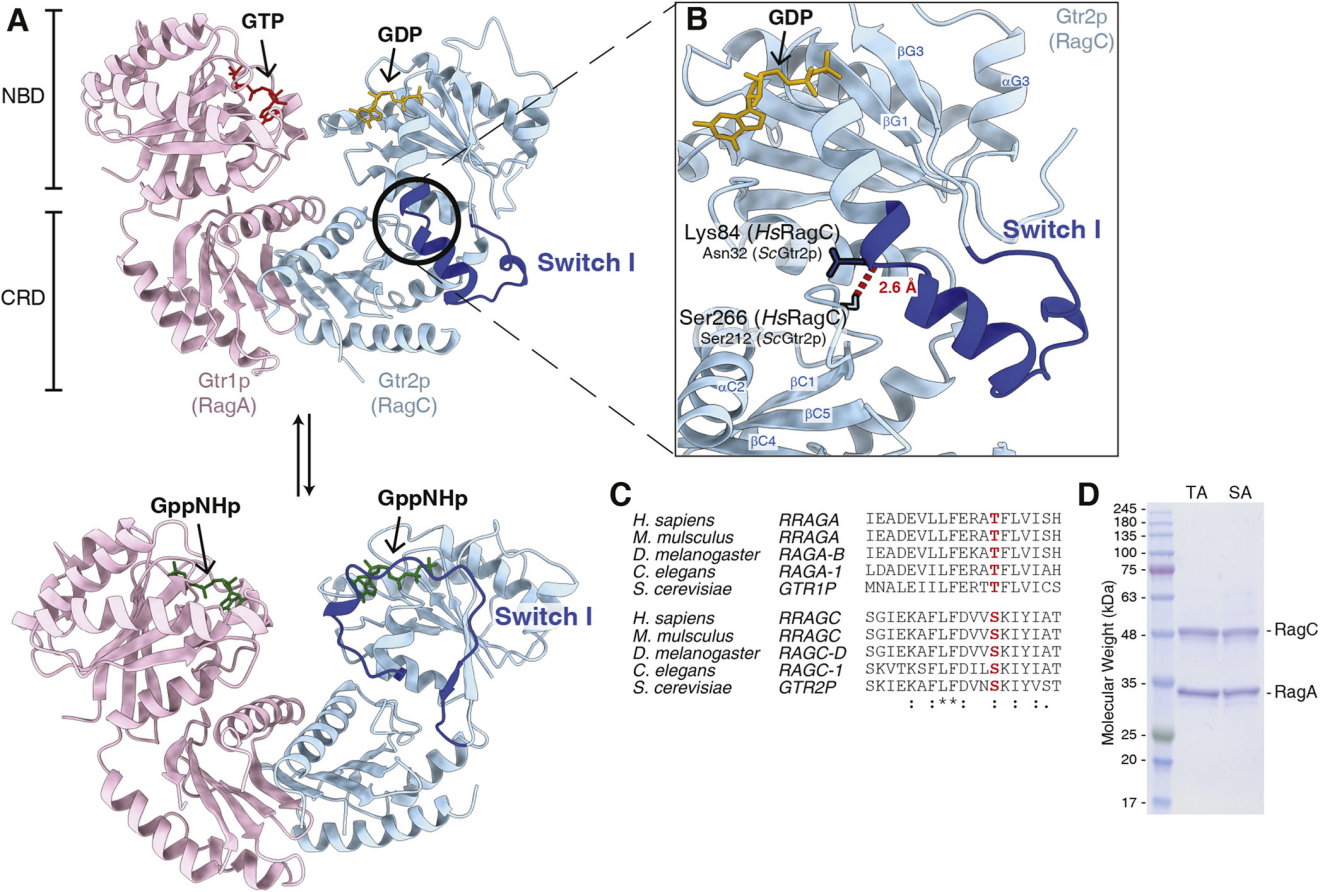
**Identification of an interdomain hydrogen bond in the Rag GTPases.***A*, structural models for the Rag homolog in yeast, Gtr1p-Gtr2p, in two nucleotide-loading states. Gtr2p (RagC) is GDP bound in the *upper panel*, with the Switch I region contacting the CRD (PDB: 4ARZ). When bound to GppNHp (*lower panel*), the Switch I region is seen bound to the nucleotide in the upward confirmation (PDB: 3R7W). *B*, zoomed-in view of the Switch I region of Gtr2p in the GDP-bound state. The interdomain hydrogen bond (*red dashed line*) extends from *Sc*Ser212 (Ser266 of *Hs*RagC) to *Sc*Asn32 (Lys84 of *Hs*RagC) and is responsible for holding the Switch I region in the downward conformation. *C*, sequence conservation of Thr210 of RagA and Ser266 of RagC. Thr210/Ser266 is conserved across diverse lineages. *D*, purified mutant Rags heterodimer assessed by SDS-PAGE and Coomassie Blue staining. CRD, C-terminal roadblock domain; *Hs*, *Homo sapiens*; SA, RagA–RagC(S266A); *Sc*, *Saccharomyces cerevisiae*; TA, RagA(T210A)–RagC.

To identify structural elements that maintain the nucleotide-loading states of the Rag GTPases, we compared the crystal structures of the Rag homolog in yeast, Gtr1p–Gtr2p, at different nucleotide-loading states (9, 10), as these two high-resolution structures presented the clearest atomic details of the dramatic conformational changes of the Switch I region upon binding to GTP *versus* GDP (Fig. 1*A*, upper panel). Upon GDP binding, Switch I of RagC forms a continuous alpha helix, which is extended downward and away from the nucleotide-binding pocket. It is tethered to the CRD in an inactive conformation (Fig. 1*A*, upper panel). In the presence of GTP, however, the Switch I region is released from the CRD and flips up-and-over the nucleotide pocket, acting as a lid to bind the nucleotide (Fig. 1*A*, lower panel). We noticed that, only in the GDP-bound state, a hydrogen bond is formed between Switch I of the NBD and CRD (Fig. 1*B*), specifically by the hydroxyl group on the side chain of Ser266 on the CRD of RagC, with the backbone carbonyl of Lys84 of Switch I on the NBD of RagC. The hydroxyl group on the side chain of Ser266 of RagC is conserved across lineages from yeast to human and so is on the corresponding residue, Thr210, on the RagA side (Fig. 1*C*). We therefore asked whether the tethering of the Switch I region to the CRD is required to stabilize the nucleotide-loading state of the Rag GTPases, specifically in their GDP-loaded state.

To probe the consequence of disrupting the hydrogen bond between Switch I and the CRD, we eliminated the hydrogen donor on the CRD by mutating Ser266 of RagC, or Thr210 of RagA, to an alanine residue. We coexpressed RagA and RagC in bacteria and purified these proteins by affinity column, ion exchange, and size-exclusion chromatography. High-quality protein complexes were deemed suitable for further biochemical characterization (Fig. 1*D*).

### RagA(T210A) and RagC(S266A) mutations do not affect the binding of nucleotides

**Figure 2:**
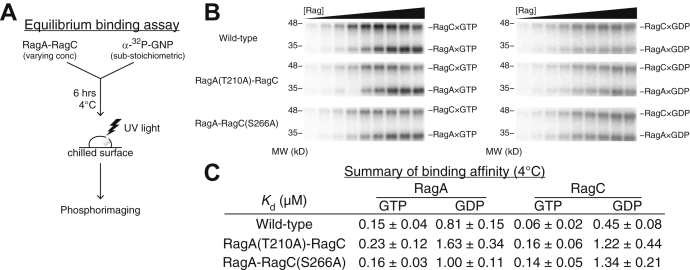
**RagA(T210A) and RagC(S266A) mutations do not affect the binding of nucleotides.***A*, experimental setup for the equilibrium binding assay used to assess nucleotide affinity to individual Rag GTPase subunit. *B*, SDS-PAGE gel to assess nucleotide binding as a function of Rag GTPases concentration. GTP binding (*left*) and GDP binding (*right*) measure the dissociation constants (*K*_d_) of nucleotides and the Rag GTPase heterodimer. *C*, summary of dissociation constants (*K*_d_) of nucleotides to the WT and mutant Rag GTPase heterodimer at 4 °C. Experiments were performed three times, and the mean ± SD was reported.

We first measured the binding affinity of guanine nucleotides to RagA(T210A)–RagC and RagA–RagC(S266A) and compared it to that of WT Rag GTPases. We reasoned that because the mutation was not introduced in the NBDs, the binding affinity of guanine nucleotides to individual Rag subunits should not change. To differentiate nucleotide binding to Rag subunits, we adapted a previously established crosslinking approach (11). Here, we incubated radioactively labeled GTP or GDP with the Rag GTPase heterodimer and irradiated the reaction mixture at equilibrium with 260-nm UV light, to induce nonspecific, zero-distance crosslinking between the bound nucleotide and the corresponding subunit (Fig. 2*A*). The binding can then be differentiated by SDS-PAGE as Rag subunits have distinct molecular weights and thus migrate at different positions (Fig. 2*B*). Using this approach, we measured the binding affinity of GTP and GDP to the mutants. Consistent with our prediction, the dissociation constants (*K*_d_) of GTP and GDP are within 2- to 3-fold of that of WT Rag GTPases (Fig. 2*C*), suggesting the nucleotide-binding pockets of the mutants remain intact and functional. Furthermore, considering the cellular concentration of GTP (∼500 μM) and GDP (∼150 μM) (12), which is much higher than the *K*_d_, the nucleotide-binding pockets of these mutants will likely be constantly occupied, similar to WT Rag GTPases.

### The Switch I–CRD hydrogen bond stabilizes the nucleotide-loading state of the Rag GTPases

To maintain a defined functional state, the Rag GTPase heterodimer has evolved mechanisms that allow for communication between the two subunits to stabilize a single-GTP–loaded state. When one subunit binds GTP and the other binds GDP, the Rag GTPase heterodimer resides in a relatively stable configuration as (1) the intrinsic GTP hydrolysis happens with a half-life of ∼50 h at 25 °C (*k*_cat_ ∼ 0.00022 min^−1^) and (2) the GTP-bound subunit will inhibit a second GTP from binding to the other subunit (11). In contrast, when both Rag subunits are forced to bind GTP, the dual-GTP–loaded state will stimulate the hydrolysis rate of the later-bound GTP molecule by ∼15-fold (11). Because the interdomain hydrogen bond we identified above can only form when a Rag subunit is loaded with GDP, we considered whether it might participate in maintaining the oppositely nucleotide-loaded state. To test this hypothesis, we carried out GTP hydrolysis experiments as detailed below.

**Figure 3:**
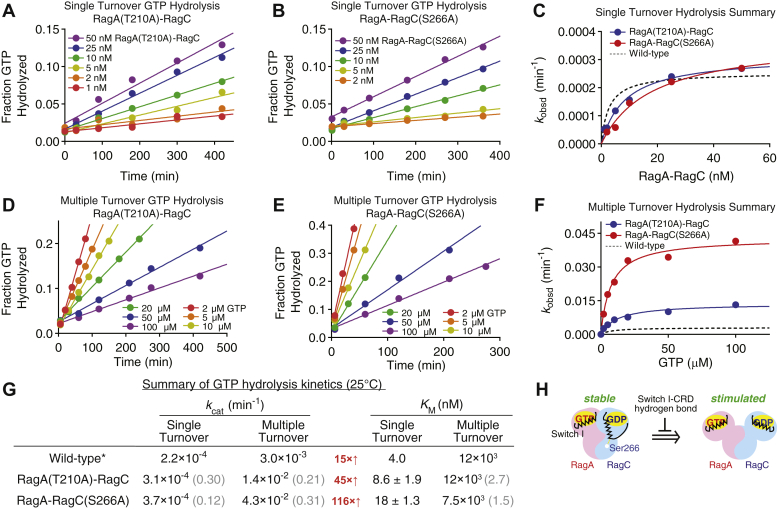
**The Switch I–CRD hydrogen bond stabilizes the nucleotide-loading state of the Rag GTPases.***A* and *D*, single-turnover (*A*) and multiple-turnover (*D*) hydrolysis experiments for the RagA(T210A)–RagC mutant. Quantification of ɑ-^32^P-GDP as a function of time returns the observed rate constant (*k*_obsd_) at various Rag GTPase (*A*) or GTP (*D*) concentrations for the RagA(T210A)–RagC mutant. *B* and *E*, single-turnover (*B*) and multiple-turnover (*E*) hydrolysis experiments for the RagA–RagC(S266A) mutant. Quantification of ɑ-^32^P-GDP as a function of time returns the observed rate constant (*k*_obsd_) at various Rag GTPase (*A*) or GTP (*D*) concentrations for the RagA–RagC(S266A) mutant. *C* and *F*, plotting *k*_obsd_*versus* the Rag GTPase or GTP concentration yields the Michaelis–Menten curve, which is used to calculate *k*_cat_ and *K*_M_ for the single-turnover (*C*) and multiple-turnover (*F*) reaction conditions. *G*, summary of hydrolysis kinetics for the WT and mutant Rag GTPases at 25 °C. The *asterisk* denotes WT data were taken from reference (11). Experiments were performed three times, and the mean ± S.D. was reported. *H*, model depicting the role of the Switch I–CRD hydrogen bond. Disrupting this contact results in the GDP-bound subunit to resemble the GTP-bound confirmation, resulting in stimulated hydrolysis. CRD, C-terminal roadblock domain.

We first performed single-turnover GTP hydrolysis experiments with the two mutants to probe the intrinsic hydrolysis rate of a single subunit (Fig. 3, *A* and *B*). Here, an excess amount of Rag GTPases was incubated with a trace amount of radioactively labeled GTP, and the hydrolysis kinetics were monitored against time. Under this setup, only one subunit of the heterodimer has one chance of hydrolyzing one round of GTP. We found that both mutants displayed very similar hydrolysis kinetics to WT Rag GTPases (Fig. 3*C*, compared with the dashed line). The *k*_cat_ values are within 2-fold of that of WT Rag GTPases, and *K*_M_ remains in low nanomolar range, suggesting the binding and hydrolysis of individual Rag subunits are not affected by the mutation (summarized in Fig. 3*G*). These results corroborate our previous binding assay that suggests individual nucleotide-binding pockets remain intact and functional.

To probe the effect of impairing the Switch I–CRD hydrogen bond on the nucleotide-loading state of the Rag GTPases, we performed a multiple-turnover hydrolysis assay, in which a saturating amount of GTP was added to a small amount of Rag GTPases, to force both subunits to bind to GTP and undergo multiple, successive rounds of GTP hydrolysis. For WT Rag GTPases, *k*_cat_ in a multiple-turnover setup is 15-fold higher than that in a single-turnover setup, suggesting when both subunits bind GTP, the Rag heterodimer tends to resolve the unstable ^GTP^RagA–RagC^GTP^ state by hydrolyzing GTP on one subunit (11). With the mutants, we observed a dramatic increase of *k*_cat_ (Fig. 3, *D* and *E*). Compared with the single-turnover condition, the stimulation for RagA(T210A)–RagC becomes 45-fold and for RagA-RagC(S266A) is increased to an even greater extent of 116-fold (Fig. 3, *F* and *G*). These results suggest that in the absence of the interdomain hydrogen bond, the Switch I motif is no longer tethered to the CRD in the downward conformation (GDP-bound conformation) and therefore tends to mimic the GTP-bound conformation. As a consequence, the Rag GTPase heterodimer is pushed more frequently to the dual-GTP–loaded state, resulting in faster *k*_cat_ only in multiple-turnover conditions (Fig. 3*H*, model).

### Directional intersubunit communication requires the Switch I–CRD hydrogen bond

**Figure 4:**
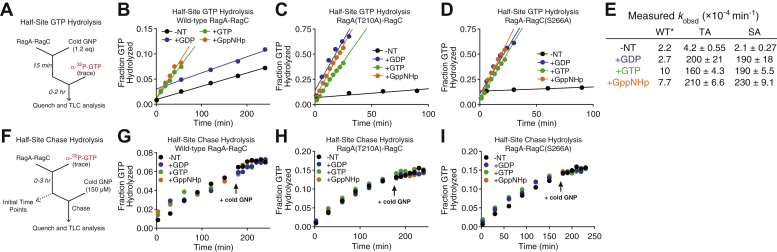
**Directional intersubunit communication requires Switch I–CRD hydrogen bond.***A* and *F*, experimental setup for the half-site (*A*) and half-site chase (*F*) hydrolysis assays used to assess the directionality of intersubunit communication. *B*–*D*, half-site GTP hydrolysis in the presence of prebound GDP (*blue*), GTP (*green*), or GppNHp (*orange*) for WT Rag GTPases (*B*), RagA(T210A)–RagC (*C*), and RagA–RagC(S266A) (*D*). All three nucleotides stimulate the hydrolysis rate similarly, as compared against no preloaded nucleotides (*black*). *E*, summary of half-site hydrolysis rates for the Rag GTPase mutant heterodimers at 25 °C. The *asterisk* denotes WT data were taken from reference (11). Experiments were performed three times, and the mean ± S.D. was reported. *G*–*I*, half-site hydrolysis chase with addition of GDP (*blue*), GTP (*green*), or GppNHp (*orange*) at 175 min for WT Rag GTPases (*G*), RagA(T210A)–RagC (*H*), and RagA–RagC(S266A) (*I*). The observed hydrolysis rate of the prebound, radioactively labeled nucleotide is unchanged upon addition of nucleotide to the second subunit. -NT (*black*) refers to no addition of nucleotides. CRD, C-terminal roadblock domain; SA, RagA–RagC(S266A); TA, RagA(T210A)–RagC.

Intersubunit crosstalk within the Rag GTPase heterodimer is unidirectional: The GTP-bound subunit will inhibit the other subunit from binding a second GTP, and in case of dual binding, trigger the hydrolysis, which ultimately secures the single-GTP loaded state (11). As our results suggest that the Switch I–CRD hydrogen bond stabilizes the GDP-loaded state, we determined whether intersubunit crosstalk requires such stabilization. We tested this hypothesis using a half-site GTP hydrolysis assay (Fig. 4*A*). In this assay, the heterodimer is first preloaded with a single GTP or GDP, followed by the addition of radioactively labeled GTP, which ensures that radioactively labeled GTP can only occupy the second subunit. Therefore, the measured apparent rate of hydrolysis will show how the Rag GTPase heterodimer handles the second GTP when it is prebound with a defined nucleotide. In the case of WT Rag GTPase heterodimer, when it is preloaded with GTP or GppNHp, the hydrolysis rate on the second subunit was increased by 5-fold (Fig. 4*B*, summarized in Fig. 4*E*). Conversely, preloading the WT heterodimer with GDP did not increase the rate of hydrolysis of the second subunit (Fig. 4*B*, summarized in Fig. 4*E*). To our surprise, when we carried out similar experiments with the mutants that have a defective Switch I–CRD hydrogen bond, prebound GTP or GppNHp stimulated hydrolysis of the later-bound GTP to a much higher level (∼50-fold, Fig. 4, *C* and *D*). Moreover, even when the Rag GTPase mutants were preloaded with GDP, later-bound GTP hydrolysis was still stimulated at a similar rate (Fig. 4, *C* and *D*, comparing +GTP and +GppNHp with +GDP), which is in sharp contrast with WT Rag GTPases (Fig. 4*E*). These results strongly suggest that the hydrogen bond between Switch I and the CRD is necessary to maintain the GDP-loaded conformation. Without it, Switch I may adopt a GTP-bound conformation even when the subunit actually binds GDP, resulting in faster hydrolysis of GTP by the other subunit and destabilization of the nucleotide-loading state.

To probe the effect of the interdomain hydrogen bond in the GTP-loaded state, we performed a half-site hydrolysis chase (11). Here, we preloaded the Rag GTPase heterodimer with a trace amount of radioactively labeled GTP and monitored its hydrolysis in the presence of an excess amount of unlabeled nucleotides as a chase (Fig. 4*F*). Interestingly, regardless of the “chase” nucleotides, prebound GTP was hydrolyzed at a constant, slow speed, similar to that of WT GTPases (Fig. 4, *G*–*I*). These results suggest the interdomain hydrogen bond is dispensable when the Rag GTPase binds GTP and are also consistent with the observation that the Switch I–CRD hydrogen bond only forms in the GDP-loaded state. Taken together, the half-site hydrolysis and half-site chase experiments further corroborate the functional role of the Switch I–CRD hydrogen bond in stabilizing the GDP-loaded state of the Rag GTPase.

### Interdomain hydrogen bonding is essential for transmitting amino acid signals

Regulation of the mTORC1 pathway is mediated in part by the recruitment or rejection of mTORC1 to the lysosomal surface *via* the interaction with the RagA–RagC heterodimer. In the presence of abundant nutrients, RagA is loaded with GTP and RagC is loaded with GDP (^GTP^RagA–RagC^GDP^). This nucleotide-loading configuration results in a conformation that favorably binds mTORC1 as it provides an optimal interaction surface with Raptor, a subunit of mTORC1. Conversely, in the absence of sufficient nutrients, the nucleotide-loading state is reversed (^GDP^RagA–RagC^GTP^) and the interaction of RagA–RagC with Raptor is weakened, so as to release mTORC1 into the cytosol. Considering the sensitivity of mTORC1 to the nucleotide-loading state of the Rag GTPases, we reasoned that, if the interdomain hydrogen bond is to stabilize the GDP-loaded state of a Rag subunit, impairment would cause unfaithful mTORC1 signaling in cells. For example, if the RagA(T210A) mutation destabilizes the RagA^GDP^ state, it will reduce the population of ^GDP^RagA–RagC^GTP^ (inactive form) and therefore cause hyperactivation of mTORC1.

**Figure 5:**
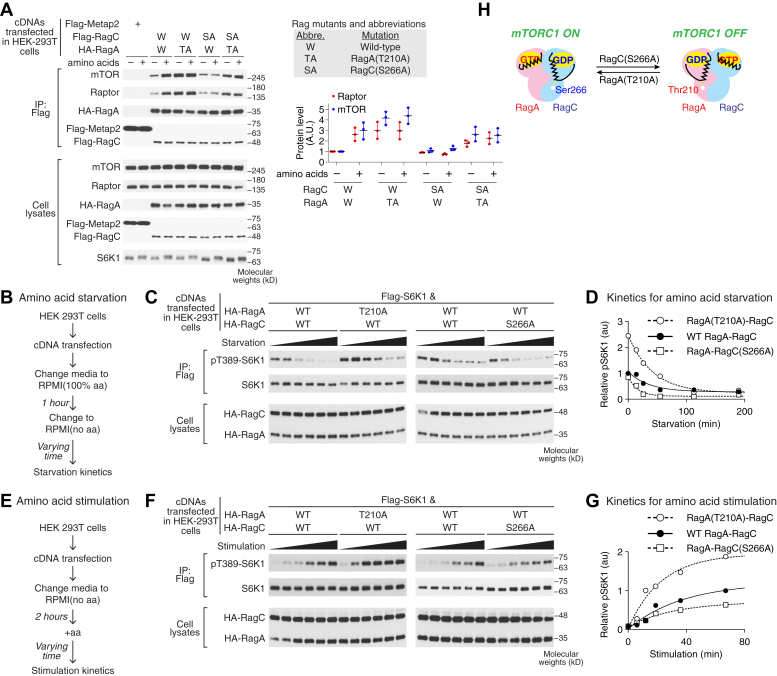
**Interdomain hydrogen bonding is essential for transmitting amino acid signals.***A*, coimmunoprecipitation of Rag GTPase heterodimer with the mTORC1 components, mTOR and Raptor, in the presence or absence of amino acids. Disrupting the Switch I–CRD hydrogen bond had opposite effects on the amounts of mTORC1 components immunoprecipitated depending on the subunit mutated. This experiment was repeated three times, and a representative was shown. Quantification of the Western blots was performed using LI-COR Odyssey imaging system, and the band intensity was normalized to the WT, minus amino acid condition. The mean ± S.D. from three repeats was reported. *B* and *E*, experimental setup for the amino acid starvation (*B*) and stimulation (*E*) assays conducted in HEK293T cell culture. *C* and *F*, effects of the interdomain mutations on the ability of cells to respond to amino acid starvation (*C*) and stimulation (*F*), as determined by quantifying the abundance of a downstream phosphorylation site of mTORC1, pThr389-S6K1. For this set of amino acid starvation experiment (*C*), the six time points were taken at 0, 14.2, 26.2, 55.5, 113, and 190 min. For this set of amino acid stimulation experiment (*F*), the six time points were taken at 0, 6.3, 12.2, 19.3, 36.8, and 67.3 min. *D* and *G*, mTORC1 activity assessed by the phosphorylation of downstream target, pThr389-S6K1, plotted as a function of time. The Rag GTPase mutations interfere with the ability of the heterodimer to respond adequately to amino acid starvation (*D*) and stimulation (*G*). The apparent rate constants for mTORC1 downregulation (*D*) are 0.028 ± 0.003 min^−1^ for WT Rag GTPases, 0.024 ± 0.002 min^−1^ for RagA(T210A)–RagC, and 0.061 ± 0.010 min^−1^ for RagA–RagC(S266A). The apparent rate constants for mTORC1 upregulation (*G*) are 0.027 ± 0.003 min^−1^ for WT Rag GTPases, 0.044 ± 0.007 min^−1^ for RagA(T210A)–RagC, and 0.031 ± 0.003 min^−1^ for RagA–RagC(S266A). Experiments were performed three times, and the mean ± SD of the apparent rate constants was reported. *H*, model depicting the role of the interdomain hydrogen bond responsible for tethering the Switch I region to the CRD in the GDP-bound state. Disrupting this stabilizing bond leads to distortions in the ability of the heterodimer to respond to changes in nutrient availability within the cell. CRD, C-terminal roadblock domain; mTORC1, mechanistic target of rapamycin complex 1.

To test the hypothesis above, we first assessed the role of the interdomain hydrogen bond on transmitting amino acid signals by probing the interaction between the Rag GTPases and mTORC1 using a coimmunoprecipitation (co-IP) assay. We transiently coexpressed one of the two mutants with the WT counterpart in HEK293T cells (Fig. 5*A*), so we can probe the amount of mTORC1 that coimmunoprecipitates with the Rag heterodimer in the absence and presence of amino acids. When WT Rag GTPases were expressed in HEK293T cells, we detected their interaction with mTOR and Raptor, both of which are mTORC1 subunits (Fig. 5*A*, lanes W/W). mTORC1–Rag interaction is regulated by amino acids, as a higher amount of mTORC1 is associated with the Rag GTPases in the presence of amino acids. However, when we impaired the interdomain hydrogen bond on RagA by expressing the RagA(T210A)–RagC mutant, a higher amount of mTOR and Raptor coimmunoprecipitates with this mutant, suggesting a more favorable interaction with mTORC1 (Fig. 5*A*, lanes TA/W). Moreover, this interaction responds less well to differences in amino acid availability, suggesting this mutant blunts the ability to switch between functional states. In contrast, the RagA–RagC(S266A) mutant interacted less stably (Fig. 5*A*, lanes W/SA), while coexpression of both mutants partially rescued the defect (Fig. 5*A*, lanes TA/SA). These results are consistent with our hypothesis that the interdomain hydrogen bond is crucial in stabilizing the functional state of the Rag GTPase heterodimer. Without such stabilization effect, the Rag GTPases tend to fall out of the original nucleotide-loading state, resulting in altered mTORC1 interaction (Fig. 5*H*, model).

We then assessed the ability of these Rag mutants to respond to changes in nutrient availability by measuring a downstream phosphorylation site of mTORC1, pT389-S6K1, in HEK293T cells. To assess the ability of the mutant to deactivate mTORC1, a starvation time course was applied (Fig. 5*B*). Cells were first treated with media containing an abundance of amino acids for 1 h, followed by application of media lacking amino acids. When starved, WT RagA–RagC heterodimer mediates a gradual decrease in pT389-S6K1 (Fig. 5*C*), which reflects downregulation of the mTORC1 pathway. In the presence of RagA(T210A)–RagC, the cells were not able to respond as rapidly to amino acid starvation as indicated by the much slower decrease observed for this mutant than the WT (Fig. 5*D*). Strikingly, this mutant also displayed a heightened baseline level of pT389-S8K1 at the start of the starvation time course. Conversely, when the RagA–RagC(S266A) mutant was expressed, the abundance of pT389-S6K1 was low to start and also decreased in abundance to a greater extent than the WT (Fig. 5*D*).

Finally, we sought to determine how the presence of these mutants affects the ability of the cells to respond to amino acid stimulation (Fig. 5*E*). Here, cells were first exposed to media lacking amino acids for 1 h, followed by application of media containing amino acids. In the context of WT RagA–RagC, amino acids induce the stimulation of the mTORC1 pathway and an increase in the abundance of the downstream target pT389-S6K1. When the RagA(T210A)–RagC mutant was expressed, cells responded much more aggressively to the presence of amino acids, rising much more rapidly and to a greater extent than the WT (Fig. 5*G*). Finally, in the presence of the RagA–RagC(S266A) mutant, the cells were not able to respond as rapidly, or to the same level, as compared with WT. These results emphasize the importance of the interdomain hydrogen bond in rapid and faithful response of cells to amino acid availability (Fig. 5*H*).

## Summary

Central to the ability of cells to respond to changes in amino acid availability is the RagA–RagC heterodimer that behaves as a molecular switch for the mTORC1 pathway—inactivating it during conditions of low amino acid availability and activating it when amino acid concentrations are high. To promote mTORC1, RagA is loaded with GTP and RagC is loaded with GDP, while the reverse nucleotide-loading configuration inhibits the pathway. In this study, we identified an interdomain hydrogen bond required for maintenance of the oppositely loaded nucleotide configuration. Abolishing this interdomain hydrogen bond dysregulates the ability of the heterodimer to maintain its oppositely loaded nucleotide configuration *in vitro*, while leading to distorted responses to changes in amino acid availability in cells.

## Experimental procedures

Chemicals and Flag-M2 affinity gel were obtained from Sigma-Aldrich. ^32^P-labeled GTP was obtained from PerkinElmer. Antibodies were obtained from the Cell Signaling Technology (CST): rabbit anti-Flag: CST 2708; rabbit anti-HA: CST 3724; rabbit anti-pT389-S6K1: CST 9205; rabbit anti-S6K1: CST 2708; goat-anti-rabbit HRP-linked antibody: CST 7074.

### Protein purifications

The Rag GTPase heterodimer was purified based on a previously established protocol (11). To generate pure proteins suitable for biochemical analysis, a pCOLADuet-1 vector encoding His_8_-R_10_-SUMO-tagged RagA was coexpressed with tagless, mutant RagC in a BL21(DE3) *Escherichia coli* strain. A 16-l LB culture was induced overnight using 0.5 mM IPTG. The following day, the bacterial cells were pelleted and resuspended using 250-ml resuspension buffer (50 mM Na-Hepes, pH 7.4; 100 mM NaCl; 2 mM MgCl_2_; 2 mM DTT; 0.5 mM PMSF; 0.05% Triton; 100 μM GDP; and protease inhibitor cocktail). The resuspended cells were passed through a microfluidizer to rupture the cells, and insoluble cellular debris was cleared from the lysate *via* centrifugation. The cleared supernatant was first applied to a hand-packed nickel-nitrilotriacetic acid column. The eluted protein was concentrated and then passed over a Mono S cation exchange column that was pre-equilibrated with 90% buffer D (50 mM Na-Hepes, pH 7.4; 2 mM MgCl_2_; 2 mM DTT) and 10% buffer D^+^ (50 mM Na-Hepes, pH 7.4; 1.5 M NaCl; 2 mM MgCl_2_; 2 mM DTT). The eluted protein was concentrated, and the His_8_-R_10_-SUMO-tag was cleaved by overnight digestion using the HRV 3C protease (Pierce/ThermoFisher). After cleavage, the protein was subjected to a second round of Mono S purification to remove the cleaved tag from the mixture. The Rag heterodimer was then applied to a Mono Q anion exchange column that was pre-equilibrated with 90% buffer D and 10% buffer D’ (50 mM Na-Hepes, pH 7.4; 1 M NaCl; 2 mM MgCl_2_; 2 mM DTT). The eluted protein was stripped by 20 mM EDTA, concentrated, and finally applied to a HiLoad 16/60 Superdex 200 gel-filtration column that was pre-equilibrated in gel filtration buffer (50 mM Na-Hepes, pH 7.4, 100 mM NaCl, 1 mM EDTA, 2 mM DTT). Glycerol (5%) was added to the final, concentrated product and was flash-frozen and stored at −80 °C until ready for biochemical analysis.

### Equilibrium binding assay

All of the biochemical assays described below were conducted in assay buffer (50 mM Na-Hepes, pH 7.4; 100 mM KOAc; 2 mM MgCl_2_; 2 mM DTT; and 0.1% CHAPS).

The binding affinity of nucleotides was measured using a previously established protocol (11). Briefly, increasing concentrations of the Rag GTPases, ranging from 5 nM to 5 μM, were incubated with trace amounts of ^32^P-labeled GTP or GDP on ice for 6 h to reach equilibrium. After that, the reaction was directly spotted onto a chilled metal block, and the mixture was irradiated with 260-nm UV light to induce zero-distance crosslinking between the nucleotide and the bound subunit. The resulting reaction products were analyzed by SDS-PAGE, and binding was visualized and quantified using a Typhoon scanner. The signal was fit to a single-site binding equation in GraphPad Prism to calculate the *K*_d_ of nucleotide binding to each subunit.

### GTP hydrolysis assay

Kinetic analyses were performed using established protocols (11). Single-turnover assays were carried out using ∼0.5 nM of ^32^P-labeled GTP with increasing amount of Rag GTPase heterodimer, ranging from 1 nM to 50 nM. Time points were taken to trace the reaction process. The quenched reaction time points were then analyzed using cellulose 300 PEI TLC plates and TLC running buffer (1 M formic acid and 0.5 M LiCl). The plates were imaged using a Typhoon scanner, and the ratio of ^32^P-labeled GDP by-product to ^32^P-labeled GTP starting material was quantified to calculate the rate constant (*k*_obsd_). The observed rate constants were fit against Rag GTPases concentration to obtain *k*_cat_ and *K*_½_ values. For the multiple-turnover assays, a fixed amount of Rag GTPase heterodimer (2 μM) was mixed with increasing concentrations of cold GTP, ranging from 2 μM to 100 μM, and doped with a trace amount of ^32^P-labeled GTP. Time points were taken to trace the reaction process. The quenched time points were analyzed in exactly the same as the single-turnover measurements, and the observed rate constants were fit against GTP concentration to obtain *k*_cat_ and *K*_M_ values for the reaction.

For the half-site hydrolysis reaction, the Rag GTPases were pre-equilibrated with 1.2 equivalent molar of unlabeled nucleotides. After that, ∼0.5 nM of ^32^P-labeled GTP was added to the mixture to initiate the half-site reaction. Time points were taken and analyzed as above, and the observed hydrolysis rates were obtained by applying linear regression to the radioactive signal *versus* time. For the half-site hydrolysis chase reaction, ∼0.5 nM of ^32^P-labeled GTP was first incubated with 5 μM Rag GTPase heterodimer to start the reaction. Early time points were taken before applying a cold nucleotide chase (100 μM) to the reaction mixture, after which additional time points were recorded. The reaction progression was plotted as the radioactive signal *versus* time.

### Co-IP experiments

Co-IP experiments were performed using an established protocol (3, 11). In brief, two million HEK-293T cells were plated on a 10-cm culture dish. Twenty-four hours later, the cells were transiently transfected with cDNAs using PEI. Thirty-six hours after transfection, cells were treated with RPMI media containing, or replete of, amino acids and lysed with CHAPS lysis buffer (40 mM Na-Hepes, pH 7.4, 5 mM MgCl_2_, 10 mM Na_4_P_2_O_7_, 10 mM Na β-glycerol phosphate, 0.3% CHAPS, and protease inhibitor). Lysates were cleared *via* centrifugation, and the supernatants were then incubated with Flag-M2 affinity gel and washed with CHAPS lysis buffer supplemented with 300 mM NaCl. Immunoprecipitated proteins were denatured by SDS loading buffer, resolved by SDS-PAGE, and analyzed by immunoblotting.

Amino acid stimulation and starvation experiments were conducted based on the procedure outlined in Figure 5, *B* and *E*. Briefly, 36 h after transfection, the HEK-293T cells were treated in RPMI media (ThermoFisher) containing, or replete of, amino acids as indicated. After the initial treatment, fresh RPMI media was applied in which amino acids were either removed (starvation), or supplemented back in (stimulation), and time points were taken to monitor the mTORC1 activity as a function of time. Finally, cells were lysed in the Triton lysis buffer (40 mM Na-Hepes, pH 7.4, 5 mM MgCl_2_, 10 mM Na_4_P_2_O_7_, 10 mM Na β-glycerol phosphate, 1% Triton, and protease inhibitor) and cleared as described above, before proceeding with the co-IP. Western blots were quantified using LI-COR Odyssey imaging system.

## Data availability

All described data are contained within this article.

## Conflict of interest

The authors declare that they have no conflicts of interest with the contents of this article.

## References

[1] Liu G.Y., Sabatini D.M. (2020). mTOR at the nexus of nutrition, growth, ageing and disease. Nat. Rev. Mol. Cell Biol..

[2] Ben-Sahra I., Manning B.D. (2017). mTORC1 signaling and the metabolic control of cell growth. Curr. Opin. Cell Biol..

[3] Sancak Y., Peterson T.R., Shaul Y.D., Lindquist R.A., Thoreen C.C., Bar-Peled L., Sabatini D.M. (2008). The Rag GTPases bind raptor and mediate amino acid signaling to mTORC1. Science.

[4] Kim E., Goraksha-Hicks P., Li L., Neufeld T.P., Guan K.-L. (2008). Regulation of TORC1 by Rag GTPases in nutrient response. Nat. Cell Biol..

[5] Inoki K., Li Y., Xu T., Guan K.-L. (2003). Rheb GTPase is a direct target of TSC2 GAP activity and regulates mTOR signaling. Genes Dev..

[6] Menon S., Dibble C.C., Talbott G., Hoxhaj G., Valvezan A.J., Takahashi H., Cantley L.C., Manning B.D. (2014). Spatial control of the TSC complex integrates insulin and nutrient regulation of mTORC1 at the lysosome. Cell.

[7] Rogala K.B., Gu X., Kedir J.F., Abu-Remaileh M., Bianchi L.F., Bottino A.M.S., Dueholm R., Niehaus A., Overwijn D., Fils A.-C.P., Zhou S.X., Leary D., Laqtom N.N., Brignole E.J., Sabatini D.M. (2019). Structural basis for the docking of mTORC1 on the lysosomal surface. Science.

[8] Anandapadamanaban M., Masson G.R., Perisic O., Berndt A., Kaufman J., Johnson C.M., Santhanam B., Rogala K.B., Sabatini D.M., Williams R.L. (2019). Architecture of human Rag GTPase heterodimers and their complex with mTORC1. Science.

[9] Gong R., Li L., Liu Y., Wang P., Yang H., Wang L., Cheng J., Guan K.-L., Xu Y. (2011). Crystal structure of the Gtr1p–Gtr2p complex reveals new insights into the amino acid-induced TORC1 activation. Genes Dev..

[10] Jeong J.-H., Lee K.-H., Kim Y.-M., Kim D.-H., Oh B.-H., Kim Y.-G. (2012). Crystal structure of the Gtr1p GTP -Gtr2p GDP protein complex reveals large structural rearrangements triggered by GTP-to-GDP conversion. J. Biol. Chem..

[11] Shen K., Choe A., Sabatini D.M. (2017). Intersubunit crosstalk in the Rag GTPase heterodimer enables mTORC1 to respond rapidly to amino acid availability. Mol. Cell.

[12] Traut T.W. (1994). Physiological concentrations of purines and pyrimidines. Mol. Cell. Biochem..

